# Genomic dynamics associated with malignant transformation in IDH1 mutated gliomas

**DOI:** 10.18632/oncotarget.6189

**Published:** 2015-10-20

**Authors:** Chul-Kee Park, Inho Park, Seungmook Lee, Choong-Hyun Sun, Youngil Koh, Sung-Hye Park, Ja Eun Kim, Hongseok Yun, Se-Hoon Lee

**Affiliations:** ^1^ Department of Neurosurgery, Seoul National University Hospital, Seoul, Korea; ^2^ SD Genomics, Seoul, Korea; ^3^ Samsung SDS, Seoul, Korea; ^4^ Department of Internal Medicine, Seoul National University Hospital, Seoul, Korea; ^5^ Department of Pathology, Seoul National University Hospital, Seoul, Korea; ^6^ Division of Hematology-Oncology, Department of Medicine, Samsung Medical Center, Sungkyunkwan University School of Medicine, Seoul, Korea

**Keywords:** clonal evolution, genomic sequencing, glioma, IDH1 mutation, malignant transformation

## Abstract

The genomic mechanism responsible for malignant transformation remains an open question for glioma researchers, where differing conclusions have been drawn based on diverse study conditions. Therefore, it is essential to secure direct evidence using longitudinal samples from the same patient. Moreover, malignant transformation of IDH1-mutated gliomas is of potential interest, as its genomic mechanism under influence of oncometabolite remains unclear, and even higher rate of malignant transformation was reported in IDH1-mutated low grade gliomas than in wild-type IDH1 tumors. We have analyzed genomic data using next-generation sequencing technology for longitudinal samples from 3 patients with IDH1-mutated gliomas whose disease had progressed from a low grade to a high grade phenotype. Comprehensive analysis included chromosomal aberrations as well as whole exome and transcriptome sequencing, and the candidate driver genes for malignant transformation were validated with public database. Integrated analysis of genomic dynamics in clonal evolution during the malignant transformation revealed alterations in the machinery regulating gene expression, including the spliceosome complex (U2AF2), transcription factors (TCF12), and chromatin remodelers (ARID1A). Moreover, consequential expression changes implied the activation of genes associated with the restoration of the stemness of cancer cells. The alterations in genetic regulatory mechanisms may be the key factor for the major phenotypic changes in IDH1 mutated gliomas. Despite being limited to a small number of cases, this analysis provides a direct example of the genomic changes responsible for malignant transformation in gliomas.

## INTRODUCTION

One of the classic concepts of cancer progression includes an evolutionary process that results from stepwise mutations with sequential subclonal selection [1]. This evolutionary process in cancer is still applicable despite traditional cancer treatment strategies that involve artificial alterations in cancer-clone dynamics [2]. However, this concept did not have direct evidence until recent advances in next-generation sequencing and bioinformatics techniques allowed direct observation of this concept [3, 4]. Clonal evolution in cancer occurs through the interaction of genomic changes of advantageous driver lesions, neutral passenger lesions, and disadvantageous lesions [2]. It is accepted that the identification of driver lesions is based on increased frequency in multiple tumors compared to normal background. Furthermore, once a genetic alteration is identified as a driver in one tumor type, that event can be more reliably interpreted even if it is infrequent [2, 5]. However, the dynamics of somatic evolution in cancer is a complex process involving the interaction of the accumulation of mutations and clonal expansions. Moreover, mutagenesis in cancer cells varies by different types of genomic abnormality, ranging from small-scale aberrations to large-scale genome events. Therefore, comprehensive analysis using a polygonal approach to genetic data in longitudinal samples is essential for understanding cancer progression. Previous studies investigated clonal dynamics in cancer progression using longitudinal samples with modern sequencing technology [3, 6–14]. However, most of the studies are from hematological malignancies or systemic metastasis of solid tumors. Unexpectedly, direct evidence of genomic dynamics of malignant transformation in glioma using longitudinal samples is rare.

The literature reports that malignant transformation of low-grade gliomas (LGGs) occurs in 45% of oligodendrogliomas, 70% of oligoastrocytomas, and 74% of astrocytomas [15]. Strong evidence shows that somatic mutation of isocitrate dehydrogenase 1 (IDH1) is related to a better prognosis in LGGs [16]. On the other hand, notwithstanding a longer latent time before malignant transformation in IDH1-mutated LGGs, it has been proposed that a higher rate of malignant transformation occurred in IDH1-mutated LGGs than in wild-type IDH1 tumors [17]. However, IDH1 mutation itself may not be the driving force for malignant transformation [18]. A recent genetic study using 23 glioma patients with longitudinal samples showed that tumor progression is related to a broad spectrum of genetic changes that cannot be explained by a simple genetic event [19]. Here, we investigated 3 pairs of longitudinal samples of IDH1-mutated LGGs and malignant transformations from the 3 samples with minimal artificial treatment effect, to seek the driver genes responsible for malignant transformation. To do this, we employed a comprehensive interpretation of multiple genomic data, including analysis of chromosomal aberrations as well as whole exome and transcriptome sequencing.

## RESULTS

### Landscape view of genomic changes of longitudinal samples of LGGs

**Figure 1:**
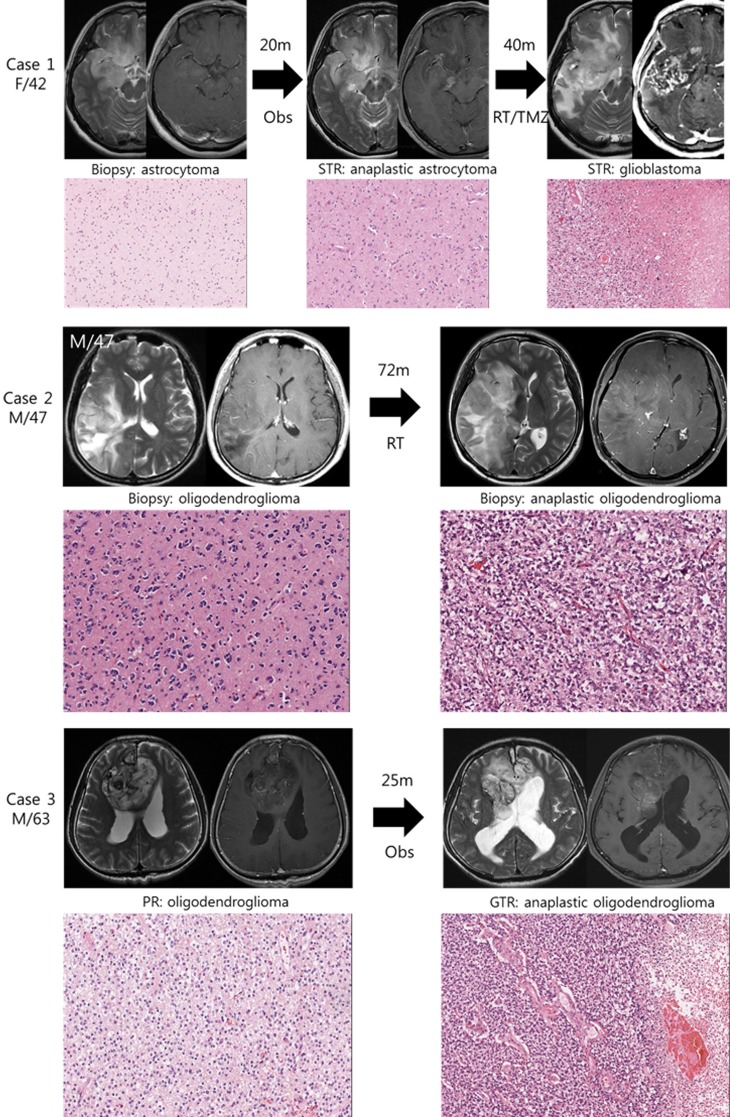
Illustrative history of patients **Case 1.** A 42-year-old female patient who was initially diagnosed with diffuse astrocytoma in right fronto-temporal area after stereotactic biopsy. At the option of the patient, she was monitored without any treatment and was in stable state for 20 months. However, enhancing area in magnetic resonance images (MRI) was developed in temporal lobe lesion, and subtotal resection was performed. The diagnosis was anaplastic astrocytoma, and she was treated with radiotherapy followed by 6 cycles of temozolomide chemotherapy. Forty months after the second surgery, progression of the disease was observed and the diagnosis of glioblastoma was confirmed after the third surgery. A pair of diffuse astrocytoma and glioblastoma samples were used in this study. **Case 2.** A 47-year-old male patient with right temporo-parietal mass was diagnosed with oligodendroglioma after stereotactic biopsy and was treated with standard radiotherapy alone. The mass was aggravated to frontal area after 72 months of stable period, and the anaplastic oligodendroglioma was confirmed after stereotactic biopsy. **Case 3.** A 63-year-old male patient with heterogeneous bifrontal mass lesion was diagnosed with oligodendroglioma after partial resection, and the remaining mass was observed over period. After 25 months of stable period, the disease started to progress and the second surgery was done. The histological diagnosis was confirmed to be anaplastic oligodendroglioma after gross total resection.

We studied 3 pairs of IDH1 mutated low grade gliomas and their high grade phenotype transformed after the lapse of time (Figure 1). The histological diagnosis were made after 2007 WHO classification criteria. Case 1 is histologically classified as astrocytoma with IDH1 mutation and intact 1p19q status, which progressed to anaplastic astrocytoma followed by glioblastoma. Case 2 and 3 are oligodendrogliomas with IDH1 mutation and 1p19q co-deletion, which progressed to anaplastic oligodendroglioma. Preservation of original tumor cells, after initial histological diagnosis by biopsy only or partial resection, enabled us to observe their clonal evolution during the progression of tumor.

**Figure 2:**
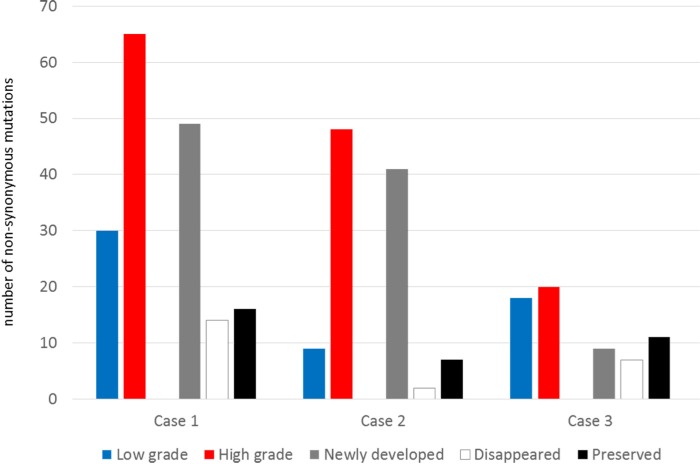
The number of non-synonymous mutations and their dynamics during the malignant transformation

To profile genomic changes during malignant transformation, whole exome sequencing (WES) was performed with pairs of low grade and high grade tumor samples, as well as with normal DNA from white blood cells. Using a paired-end sequencing strategy, nonsynonymous somatic point mutations and small insertions/deletions (indels) that change the protein amino acid sequence were identified from WES results, and gene sets were built to enable comparison between low grade and high grade counterparts in patient samples (*Dataset 1*). The number of mutated genes identified from WES is summarized in Table 1. From the standpoint of changes in the number of nonsynonymous mutations, major genetic dynamics were significantly altered during the malignant transformation process in Cases 1 and 2, while genetic changes were relatively stable in Case 3 (Figure 2). We also detected a number of somatic copy number alterations (SCNAs) within the tumor samples by using single nucleotide polymorphism (SNP) array and WES data. The segments with heterozygous deletion are of primary interest because they can be further utilized for inferring clonal dynamics of tumors. We summarize the DNA copy number alterations with the estimated allele specific copy number status and cellular fraction in *Dataset 2*. We visualized the whole-genome alterations of each sample in Circos and Manhattan-style plots for an overall view of genomic changes related to malignant transformation (Figure S1).

**Table 1:** Sequencing coverage parameters and number of genes profiled and analyzed from whole exome sequencing

	Case 1			Case 2			Case 3		
	Normal	Low grade	High grade	Normal	Low grade	High grade	Normal	Low grade	High grade
Mean depth	153	156	152	156	160	159	170	159	157
10X	91.3%	91.1%	90.5%	91.9%	91.8%	91.9%	93.1%	91.5%	91.6%
30X	83.3%	83.3%	80.8%	84.6%	83.8%	84.0%	86.3%	83.1%	83.3%
50X	75.5%	76.2%	71.8%	77.7%	76.1%	76.5%	80.1%	75.3%	75.5%
100X	56.2%	56.9%	51.5%	58.0%	57.4%	57.6%	62.5%	56.6%	56.6%
**Number of nonsynonymous somatic mutations**									
Missense	-	25	52	-	7	41	-	15	18
Start Lost	-	0	0	-	1	1		0	0
Stop Gain	-	4	4	-	0	0	-	0	0
Stop Loss	-	1	1	-	0	0	-	0	0
Codon InDel	-	0	2	-	0	0	-	0	0
Frame Shift	-	0	6	-	1	6	-	3	2
**Number of nonsynonymous mutational changes during the tumor progression**									
newly developed		49			41			9	
disappeared		14			2			7	
preserved		16			7			11	

### Clonal evolution during the malignant transformation

**Figure 3:**
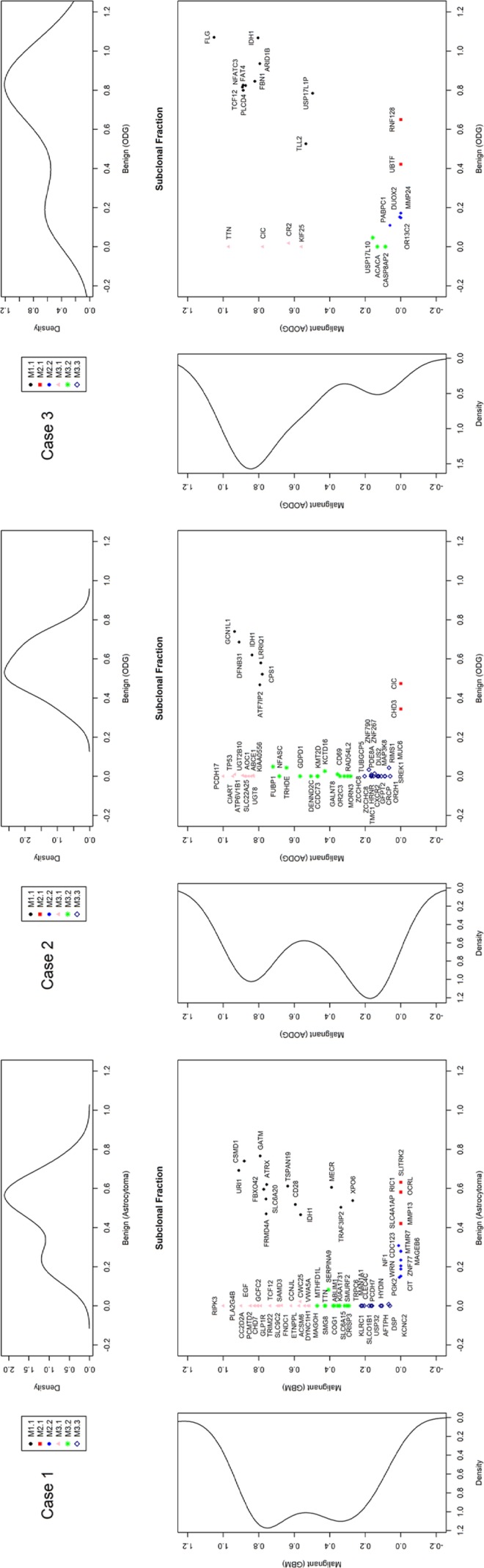
Estimated clonal fractions and their genetic signatures based on somatic point mutation allele frequency and copy number status

**Figure 4:**
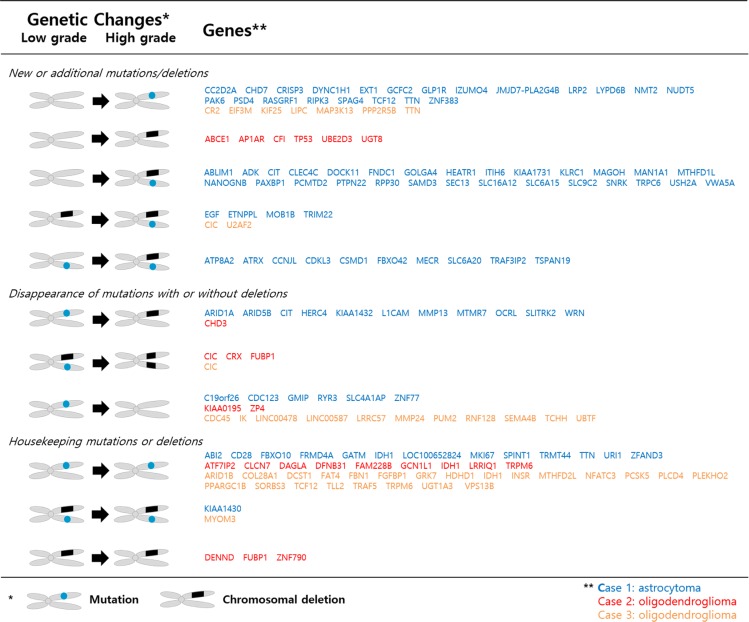
Subclone defining genes involved in genetic changes during malignant transformation

**Figure 5:**
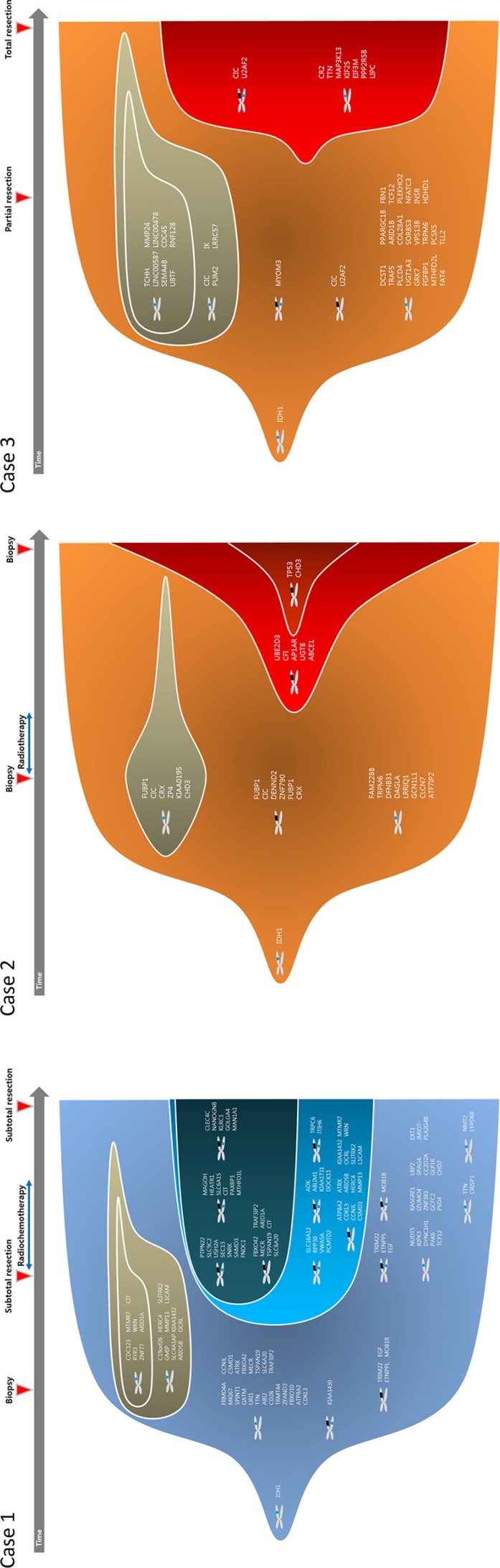
Clonal evolution from low grade to high grade gliomas

To track clonal evolution within tumors, we developed a modeling system that included a stratified grouping of SCNAs and somatic point mutations based on the estimated fraction of cancer cells harboring each alteration in a sample (Table S1). Using this modeling system, clonal fractions in tumors were estimated from SCNAs in each sample from the 3 cases (Table 2). In addition, we analyzed clonal fractions in tumors by clustering somatic point mutations based on the estimated fraction of cancer cells harboring each point mutation in a sample, which are calculated from the mutation allele frequency and copy number status of the segment containing the mutation of interest (Figure 3). Combining SCNAs and mutation analysis, lists of genes were filtered to define the subclones involving malignant transformation. The filtered genes are summarized and categorized according to their changes in status during the tumor progression (Figure 4). Collectively, we built a model for the genomic dynamics of clonal evolution during the malignant transformation in each case of IDH1-mutated glioma (Figure 5).

**Table 2:** Estimated clonal fractions of each tumor sample analyzed from somatic copy number alteration (SCNA)

Group	Clonal fraction		CNA segments
	Low grade	High grade	
***Case 1***			
S1.1	64%	84%	4q13–4q35; 11p15; 19q13
S3.1		84%	1p36; 5p15–5q35; 8p23–8p23; 8p22–8q11; 9p24–9p23; 10p12–10q26; 10p12–10q26; 11q13–11q25; 13p13–13q34; 16q11–16q24; 20q11–20q13; 22p13–22q13; Xp22-Xq28
S3.2		43%	1p36–1q44; 3p26–3p21; 6q13–6q27; 12p13–12q24; 21p13–21q22
***Case 2***			
S1.1	60%	89%	1p36–1p11; 19q11–19q13
S3.1		88%	4q12–4q24; 14q11–14q32; 17p13–17p11
S3.2		52%	18p11–18q23
***Case 3***			
S1.1	88%	90%	1p36–1p11; 19q11–19q13

### Validation of candidate genes for malignant transformation

**Figure 6:**
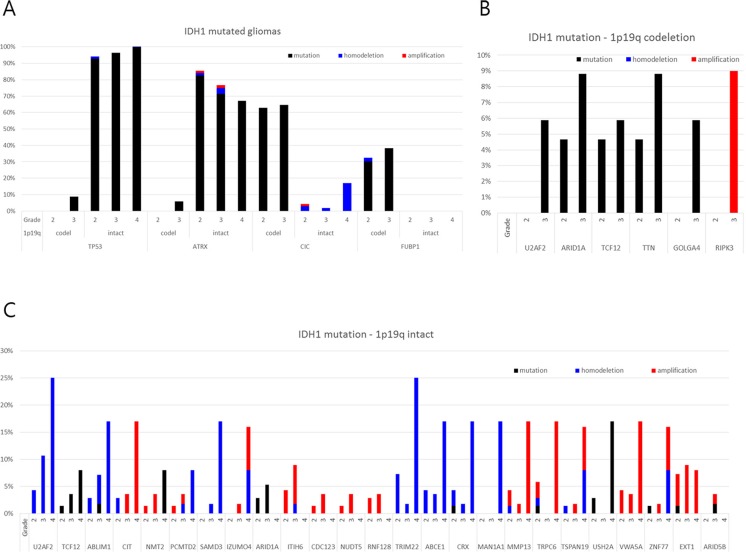
Frequency of genetic alterations of genes showing disparity according to the histological grade in the TCGA database among those with novel or additional mutations and/or copy number alterations observed in the present study

To validate genes involved in malignant transformation that were chosen for the present analysis, we used datasets from The Cancer Genome Atlas (TCGA) (https://tcga-data.nci.nih.gov/tcga/) and the cBioPortal for Cancer Genomics (http://www.cbioportal.org/public-portal/). Case sets were built from the brain lower-grade glioma (provisional) and glioblastoma (provisional) datasets. Among 262 cases from lower-grade glioma and 235 cases from glioblastoma with complete sequencing and CNA data, a total of 216 cases with an IDH1 mutation and available information about histological grade were analyzed (Table S2). We tested all the genes that showed changes during the malignant transformation in our cases. Only genes with novel or additional mutations and/or CNAs observed repeatedly in the stratified subgroup of 1p19q chromosomal status and histological grade were highlighted (Figure 6). As verified recently, TP53 and ATRX mutations were the hallmark of 1p19q intact IDH1-mutated gliomas, while CIC and FUBP1 mutations were found in 1p19q co-deleted gliomas [20–22]. However, it is worth noting that TP53 and ATRX mutations were observed only in a small fraction of 1p19q co-deleted gliomas of high grade, which implies that these types of trans-lineage mutations can contribute to the malignant transformation that was also observed in Case 2. Recent observation of changes in TP53 expression in sequential samples of oligodendrogliomas supports that the de novo TP53 mutation or the proliferation of a subset of cells with nuclear expression of TP53 could lead to tumor progression in some IDH1-mutated oligodendroglial tumors [17]. Among the genes that showed differences in incidence of mutation or CNA among grades, U2AF2 (also known as U2AF65), TCF12 (also known as HEB, HTF4 and ALF1), and ARID1A (also known as BAF250a) were commonly observed to be altered progressively in both 1p19q intact and co-deleted tumors. These genes are components of the machinery that regulates gene expression, including the spliceosome complex (U2AF2), transcription factors (TCF12), and chromatin remodelers (ARID1A).

U2AF2 had a copy number loss in case 3 from the low-grade stage and developed additional missense mutations at the high-grade stage. And in TCGA samples, 5.9% of grade 3 gliomas with IDH1 mutation/1p19q co-deletion harbored mutations in U2AF2 while no mutations were found in grade 2 gliomas with the same molecular signature (Figure 6). Moreover, in IDH1 mutation/1p19q intact gliomas subjected to TCGA, there was increase tendency of copy number loss with WHO grade (4.3%, 10.7% and 25.0% in grade 2, 3, and 4, respectively). A novel frameshift deletion in TCF12 was found in the high grade sample of case 1, and mutations were observed in 4.7% of grade 2 and 5.9% of grade 3 of IDH1 mutation/1p19q co-deletion samples in TCGA (Figure 6). Again, we observed a stepwise increase of mutation incidence with WHO grade (1.4%, 3.6%, and 8.0% in grade 2, 3, and 4) (Figure 6C). All these mutations generate either frameshifts or occur at splice-sites which suggest loss-of-function mechanisms correlating with lower expression [23]. Although a mutation in low grade phase was already present, newly developed copy number loss of ARID1A was found in the high grade sample of case 1. TCGA data shows that increase in mutation rate in high grade phenotype was observed in both 1p19q co-deleted and intact gliomas with IDH1 mutation (Figure 6). Moreover, copy number loss was accompanied by 1p19q co-deleted tumors, but there was no mutation in grade 4 GBMs as observed previously [5].

### Transcriptomal changes involved in the malignant transformation

Two pairs of tumor samples (Cases 2 and 3) were analyzed with RNA-sequencing (RNA-seq). The results of the RNA-seq analysis are summarized (Table S3), and there were minimal differences in read counts among the samples. After we quantified gene expression levels as RPKM (Reads Per Kilobase per Million mapped reads) from the RNA-seq data, we performed fold change analysis between the low grade and high grade samples for each case. To define a group of differentially expressed genes (DEGs) in each case, we used a log2 fold change cut off of > 3 (*Dataset 3* and Figure S2). Among the DEGs between low grade and high grade phenotypes in each case, HBB and HBA1 were commonly overexpressed in high grade samples of both cases. The low number of common DEGs originates from the low number of DEGs in Case 3. This implies that, although the histological diagnosis distinguishes the tumor grades, Case 3 was on track for the early phase of malignant progression at the time of the first surgery, which is also suggested by the relatively short span of time before recurrence (25 months) and the stable status of mutation frequency (Figure 2). The hierarchical clustering analysis also supports the relatively similar genomic signatures between low grade and high grade samples in Case 3 compared with those of Case 2 (Figure S3). So, we focused on Case 2 to evaluate the expression changes that are responsible for the malignant transformation. Among the overexpressed genes in the high grade phenotype, the notable genes are OLIG1, OLIG2, VGF, SOX4, SOX8, MYT1, and PDGFRA, which are known to regulate oligodendrogenesis [24]. Recent studies suggest that overexpression of these genes could be used as a representative feature of a specific subtype of glioma [25]. We performed a gene set enrichment analysis (GSEA) with the DEGs in Case 2, using C2 category gene sets in MSigDB [26, 27]. The analysis identified 7 overexpressed and 2 down-regulated gene sets in the malignant phenotype, which are significantly enriched at the nominal value of FDR q < 1.0e-10 (Table 3). Interestingly, genes that are down-regulated during differentiation of the oligodendroglial precursor (Gene set: GOBERT_OLIGODENDROCYTE_DIFFERENTIATION_DN) were reactivated during the malignant transformation, indicating that malignant transformation accompanies the restoration of stemness of the cancer cells (Figure S4) [28]. This is also supported by the overexpression of genes normally enriched in embryonic stem cells (Gene Set: BENPORATH_SUZ12_TARGETS) in the high grade phenotype (Figure S4) [29]. Identification of genes with high-CpG-density promoters bearing histone H3 dimethylation at K4 (H3K4me2) and trimethylation at K27 (H3K27me3) (Gene set: MEISSNER_BRAIN_HCP_WITH_H3K4ME3_AND_H3K27ME3) from embryonic stem-cell-derived neural precursor cells in the malignant phenotype provides further supporting evidence for the restoration of stemness (Figure S4) [30]. Interestingly, the DEG from the high grade phenotype share a common genetic signature with a proneural type of glioblastoma (Gene set: VERHAAK_GLIOBLASTOMA_PRONEURAL), which is distinguished from other glioblastomas by lower age, better prognosis, PDGFRA expression, and frequent IDH1 mutation (Figure S4) [31]. Using a recently suggested glioma classification module based on genes related to EGFR or PDGFRA expression, the PDGFRA signature became more evident with malignant transformation in Case 2 (Figure S5) [25].

**Table 3:** Gene set enrichment analysis for differentially expressed genes in low grade and high grade phenotype of case 2

Gene Set Name		# Genes in Gene Set (K)	# Genes in Overlap (k)	k/K	Enrichment score (ES)	*p*-value	FDR *q*-value
***A. Gene sets enriched with genes over-expressed in high grade phenotype***							
(a)	MILI_PSEUDOPODIA_HAPTOTAXIS_DN	668	41	0.0614	0.2736	8.04E-22	3.80E-18
(b)	GOBERT_OLIGODENDROCYTE_DIFFERENTIATION_DN	1080	50	0.0463	0.1704	4.58E-21	1.08E-17
(c)	PATIL_LIVER_CANCER	747	31	0.0415	0.2521	3.19E-12	1.07E-9
(d)	MEISSNER_BRAIN_HCP_WITH_H3K4ME3_AND_H3K27ME3	1069	47	0.0440	0.2492	5.88E-19	9.25-16
(e)	VERHAAK_GLIOBLASTOMA_PRONEURAL	210	21	0.1000	0.3899	6.36E-16	6.00E-13
(f)	REACTOME_BETA_DEFENSINS	42	11	0.2619	0.8475	9.75E-14	5.76E-11
(g)	BENPORATH_SUZ12_TARGETS	1038	43	0.0414	0.2278	1.70e-16	2.01E-13
***B. Gene sets enriched with genes underexpressed in high grade phenotype***							
(a)	BLALOCK_ALZHEIMERS_DISEASE_UP	1691	48	0.0284	−0.2388	3.69E-12	1.16E-9
(b)	LU_AGING_BRAIN_UP	262	17	0.0649	−0.3781	3.77E-10	8.09E-8

## DISCUSSION

We have performed an integrated analysis of genomic dynamics in clonal evolution during the malignant transformation and identified alterations in the machinery regulating gene expression, including the spliceosome complex (U2AF2), transcription factors (TCF12), and chromatin remodelers (ARID1A). U2AF2 is a core member of the spliceosome machinery [32], so mutations in this gene can affect the normal function of spliceosomes resulting in the formation of aberrant mature mRNAs by misunderstanding of splice site recognition [33, 34]. This kind of abnormal processing may alter the expression of multiple genes. Mutations to spliceosome genes are related to hematological malignancy and its prognosis and can act as a driver of oncogenesis in colon cancer [34–38]. Since alternative splicing is observed in extensive numbers of genes from many different types of cancer, targeting spliceosome function may unlock a novel strategy for cancer therapy [32]. TCF12 encodes a transcription factor of the basic helix-loop-helix (bHLH) E-protein family that can directly bind to E-box motfis [39, 40]. TCF12 is associated with proliferation, survival, and fate decisions in the oligodendrocyte lineage [41]. Interestingly, significant differences in TCF12 expression between 1p19q co-deleted tumors and intact tumors (higher with 1q19q codeletion) as well as among WHO grades (highest in grade 2 and lowest in grade 4) were reported previously [42]. However, whether or not TCF12 plays a role in cancer development and progression has not been determined yet except for the contradictory evidence in colorectal cancer [40, 43]. ARID1A is recurrently mutated in various cancer types [44]. ARID1A is a member consisting of SWI/SNF chromatin remodeling complexes from which disordered chromatin regulation can induce a distinct mechanism contributing to tumor development [44]. Originally, ARID1A loss was known to be an early cancer promoting event in endometriosis leading to ovarian clear cell carcinoma [45]. Evidences of discordance between expression and heterozygous mutations or loss of heterozygosity in cancer samples imply that reduced levels of ARID1A may be the contributing factor in promoting cancer [44]. Our data suggest that not only mutations, but also copy number alterations and expression of ARID1A should be investigated to confirm its oncogenic role in gliomas.

A recent sequencing based analysis of paired gliomas of low grade and their relapse has revealed that a wide spectrum of genomic dynamic exists during the tumor progression from linear clonal evolution to branched clonal evolution [19]. And they found that IDH1 was the only shared mutation among longitudinal samples in every patients [19]. That means it is difficult to identify common target gene that drive malignant transformation or tumor progression in gliomas, which is retold in the present study. It is notable that the genes of interest, drawn from comprehensive genomic analysis of malignant transformation using longitudinal samples, are regulating the components of multiple genes with diverse mechanisms involving spliceosome machinery, transcription factors, and chromatin remodelers. This suggests that alterations in genetic regulatory mechanisms may be the key factor for the major phenotypic changes in gliomas. Moreover, expression changes resulting from genomic alterations appear to activate genes associated with the restoration of stemness in cancer cells. Whether restoration of stemness is really occurring will require further investigation incorporating a large collection of longitudinal samples will provide more detailed and definite answers on this issue. The limitation of this study harbors the small number of cases, which is compensated with the incorporation of TCGA dataset in search of common gene of interest. However, a sufficient number of appropriate paired samples are needed to confirm the solid conclusion in the future. The other thing is that initial samples of low grade status might have not represented the characteristics of tumor on the whole as they were histologically diagnosed with only a spot biopsy. This issue also exhibits problem about intratumoral heterogeneity and may have act as a confounding factor for the mutational analysis.

## MATERIALS AND METHODS

### Patients and samples

A total of 3 pairs of snap-frozen samples of sequential low grade and high grade histology from 3 patients with IDH1-mutated gliomas were used for DNA and RNA extraction. DNA from white blood cells from the same patients were used as a control. This study was approved by the Institutional Review Board of Seoul National University Hospital, Seoul, Korea. Genomic DNA was extracted using the QIAamp DNA mini kit (Qiagen, Cat. No. 51304), and total RNA was extracted using RNeasy Plus Universal Mini Kit (Qiagen, Valencia, CA, USA, Cat no. 73404) according to the manufacturer's recommendations. DNA content was quantitated using the Qubit DNA quantification kit (Invitrogen, Carlsbad, CA), and DNA integrity was assessed by gel electrophoresis. Samples with an RIN (RNA Integrity Number) > 5 were selected for the study.

### Single nucleotide polymorphism array

We applied a genome-wide SNP array (Illumina HumanOmini5-Quad BeadChip, Illumina) to genomic DNA according to the manufacturer's instructions. The SNP array data was processed with GenomeStudio to generate B allele frequencies (BAF) and then applied to allele-specific copy number analysis of tumors (ASCAT) [46] to estimate tumor purity and ploidy of tumor samples.

### Whole exome sequencing

Whole exome sequencing (WES) using Agilent SureSelect Human All Exon 50Mb (Agilent Technologies Inc., Santa Clara, CA) was performed on genomic DNA, followed by sequencing with 100-bp paired end reads on the Illumina HiSeq platforms. After we aligned the raw sequencing reads to hg19 with BWA-MEM and preprocessed the initially aligned bam files using the work flow for data pre-processing steps from GATK Best Practices, we obtained final bam files with more than 150 times depth of coverage on target for all samples (Table 1).

Single nucleotide variants (SNVs) and indels were detected with three different variant callers (UnifiedGenotyper, LoFreq, and SNVer), and the resulting call-sets were filtered using false positive filters and germline filters. Recurrent cancer mutations were investigated separately to rescue mutations that could be missed by the above variant callers. Somatic variant calling was done by Fisher's exact test with *p*-value < 0.02 and odd ratio > 5.0 with read counts supporting reference allele and alternate allele at the variant position between a tumor and normal sample. Details of the somatic variant analysis pipeline are described in Figure S6.

### Whole transcriptome sequencing

RNA-seq libraries construction was performed using the manufacturer's instructions, and RNA sequencing was done on the Illumina HiSeq platform with 100-bp paired end reads. For RNA sequencing data analysis, alignment was performed by TopHat with hg19 and GENCODE version 10 (Ensembl 65), and expression profiles were analyzed using the Reads Per Kilobase per Million mapped reads (RPKM) values. Details of data analysis are described in Figure S7.

### Tumor purity and ploidy

Tumor purity and the average ploidy of the tumor samples were estimated from SNP array data by applying ASCAT [46], whose results are summarized in Table S4. The average ploidy of the tumor samples was estimated to be 1.8–2.1, except for the high grade sample from Case 2, whose ploidy was estimated to be 3.80. However, we found that the ASCAT algorithm overestimated the ploidy of the high grade sample from Case 2 because the algorithm prefers to assign integer copy numbers to segments with heterozygous deletion in chromosomes 2 and 18, even though they are only likely to be altered in just under half of the cancer cells from the sample (Figure S8). Tumor purity assessment revealed 62–91% purity, which implies the tumor samples are eligible for this study.

### Subclonal analysis

With SNP array and WES data, we could infer clonal architectures of tumor samples for a patient using the following steps. 1) We identified segments with heterozygous deletions whose size is greater than 10Mb in each tumor sample. Then, we grouped all the segments identified from all the tumor samples for a patient. The identified segments for each case is shown in *Dataset* 2.2. For each tumor sample, we calculated the fraction of a subclone harboring a segment with heterozygous deletion by utilizing two types of information: a) the normalized read count ratio between the normal and tumor sample within the segment, and b) the altered allele frequencies of germline heterozygous SNVs in a tumor sample within the segments. We qualified germline heterozygous SNVs as having allele frequencies between 0.3 and 0.7 in the normal sample. 3) Then, we infer the fraction of cells that harbor the copy number alteration on the segment of interest by using the method described in Figure S9. 4) We grouped the segments based on the inferred clonal fractions in each tumor samples, and each group represents different subclones in the tumor samples. 5) For somatic point mutations, we estimated the fraction of a subclone harboring a somatic SNV by considering the copy number status of the locus. We only considered SNVs within diploid and segments with heterozygous deletions. The estimations of cellular fraction for each somatic SNV are shown in *Dataset 1* (B. Frac and M. Frac columns in the excel sheet). And then, we grouped somatic SNVs based on the cellular fractions in tumor samples. Of note, the estimated fraction of subclones based on heterozygous deletions and on somatic SNVs is quite similar, showing the robustness of estimation of clonal architecture.

## SUPPLEMENTARY MATERIALS FIGURES, TABLES AND DATASETS








