# Supplementary material for: Genomic dynamics associated with malignant transformation in IDH1 mutated gliomas
Source: Oncotarget. 2015 Oct 20;6(41):43653–66. doi: 10.18632/oncotarget.6189 (PMC4791257; doi:10.18632/oncotarget.6189)
Supplement: Supplementary file 1 [file oncotarget-06-43653-s001.pdf]

Genomic dynamics associated with malignant transformation in IDH1 mutated gliomas

Supplementary Materials

Case 1

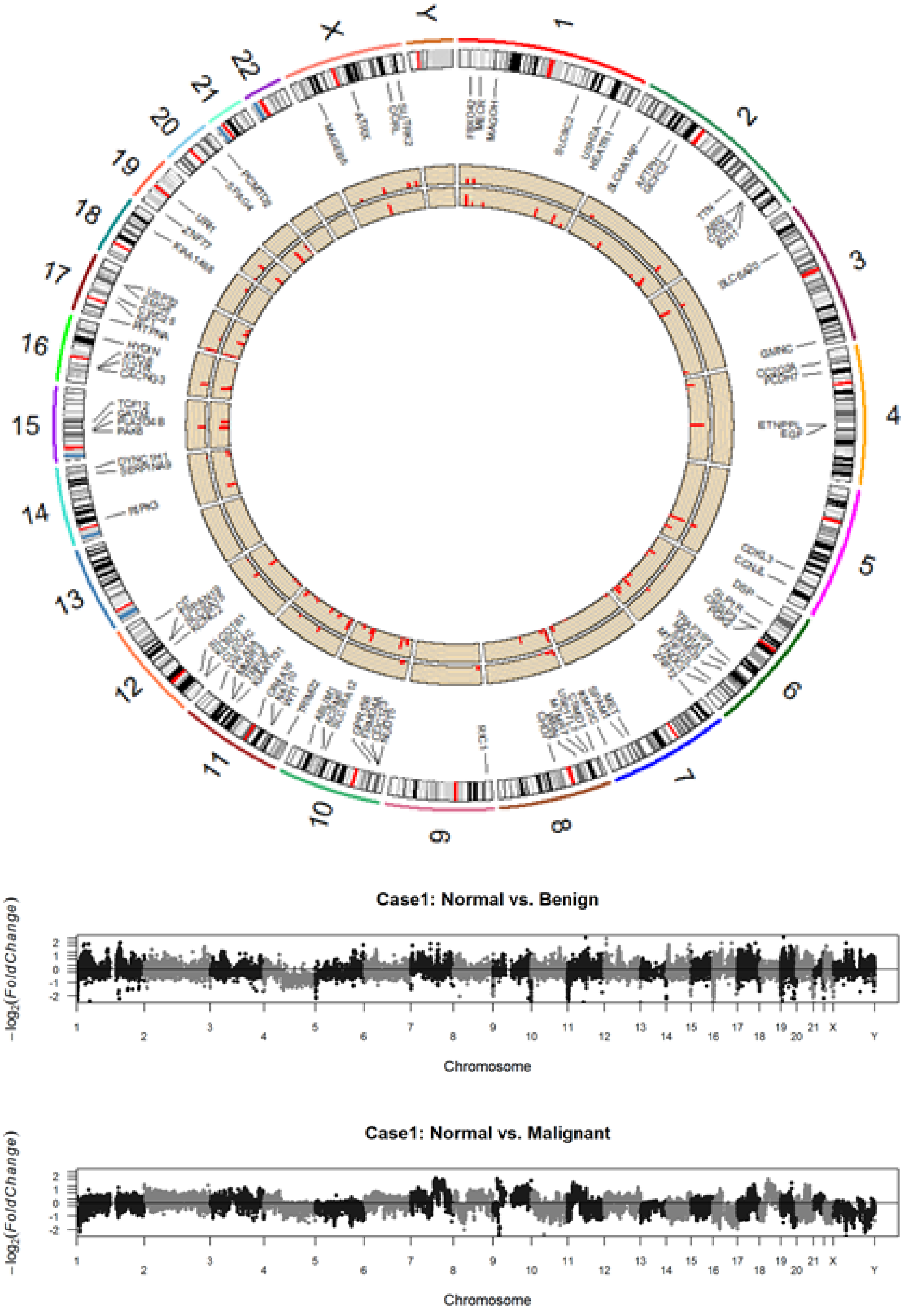

Case 2

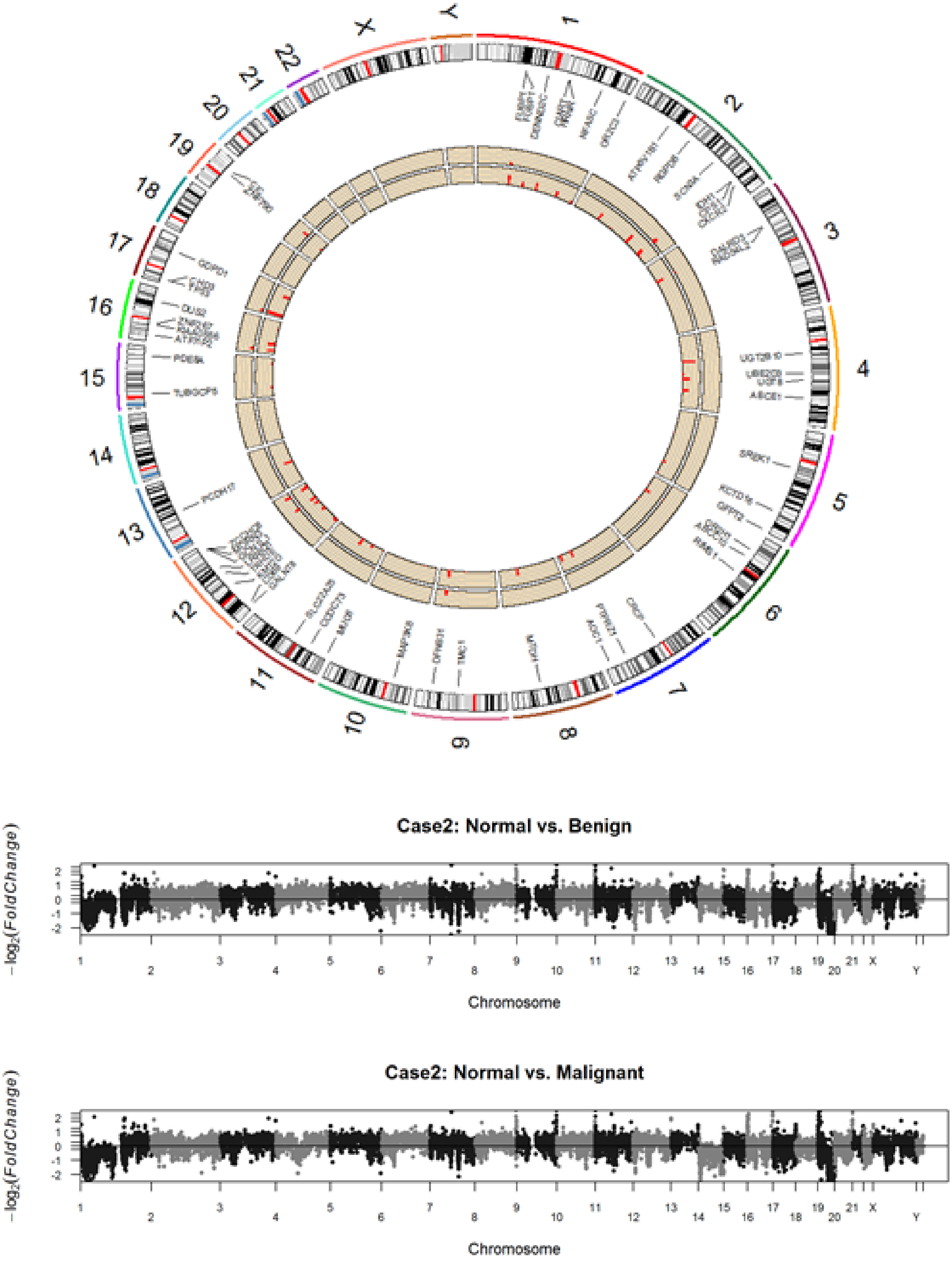

Case2: Normal vs. Benign
$-\log_2(Fold Change)$
Chromosome
Case2: Normal vs. Malignant
$-\log_2(Fold Change)$
Chromosome

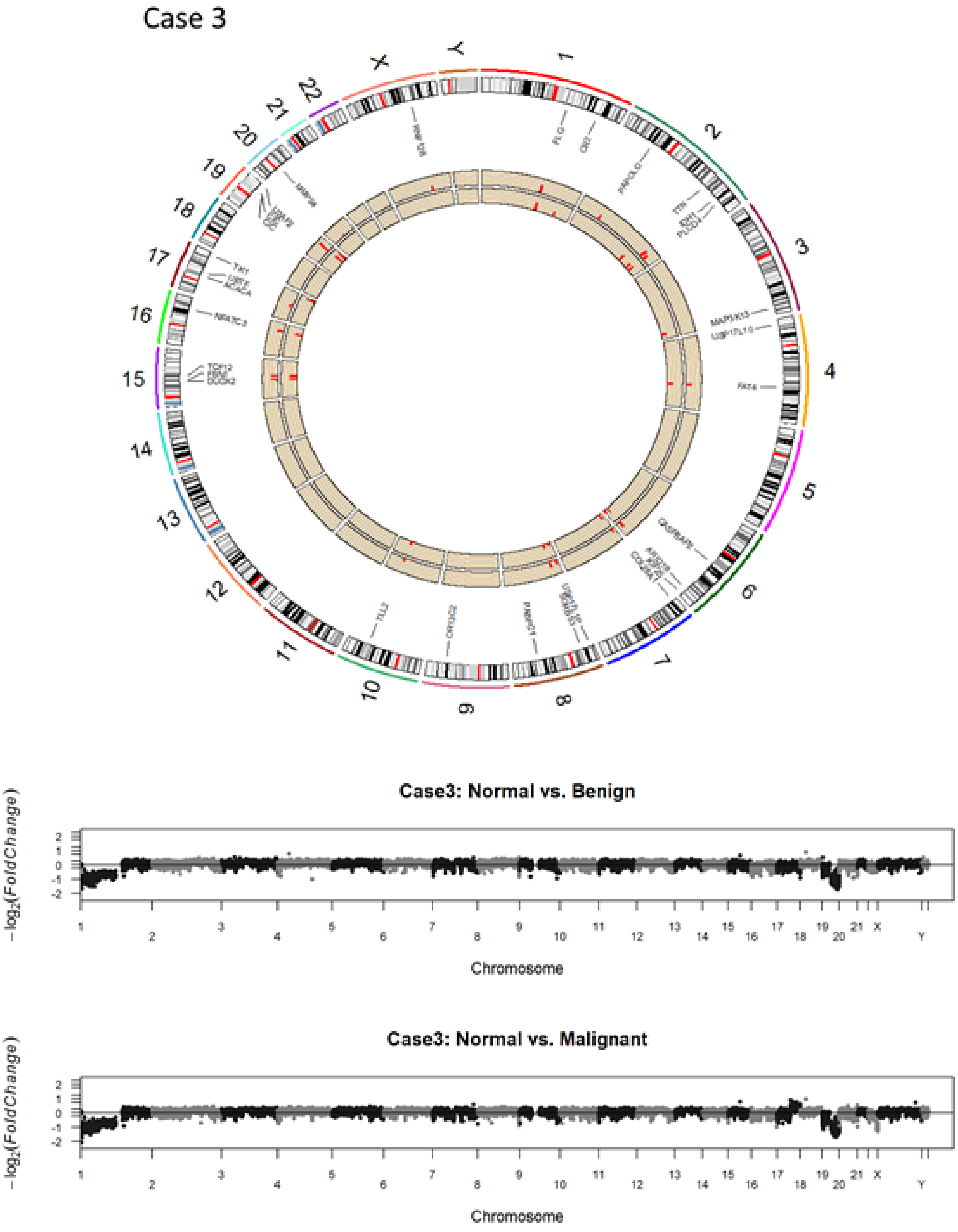

Supplementary Figure 1: The landscape view of genomic changes of each case. Genomic changes and copy number variations are presented with Circos plots and Manhattan style plots.

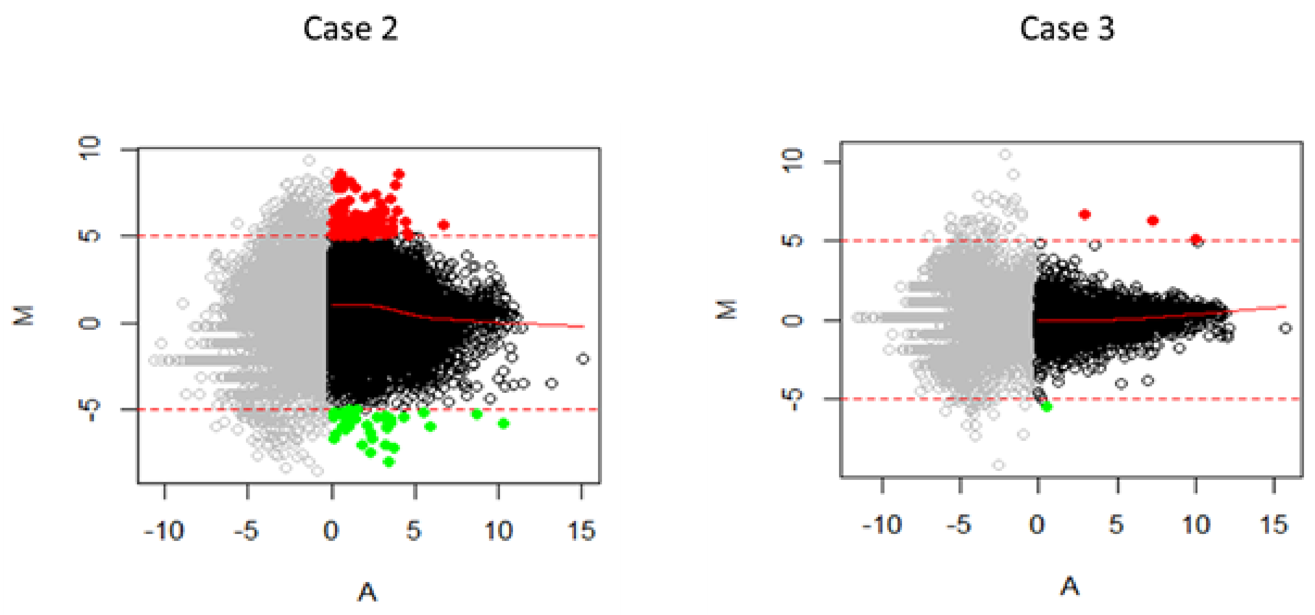

Supplementary Figure 2: MA plots for RNA-seq expression analysis of Case 2 and Case 3. A is the $(x + y)/2$, and M is the $x - y$ where x is the log2 RPKM of the benign sample, and y is that of the malignant sample.

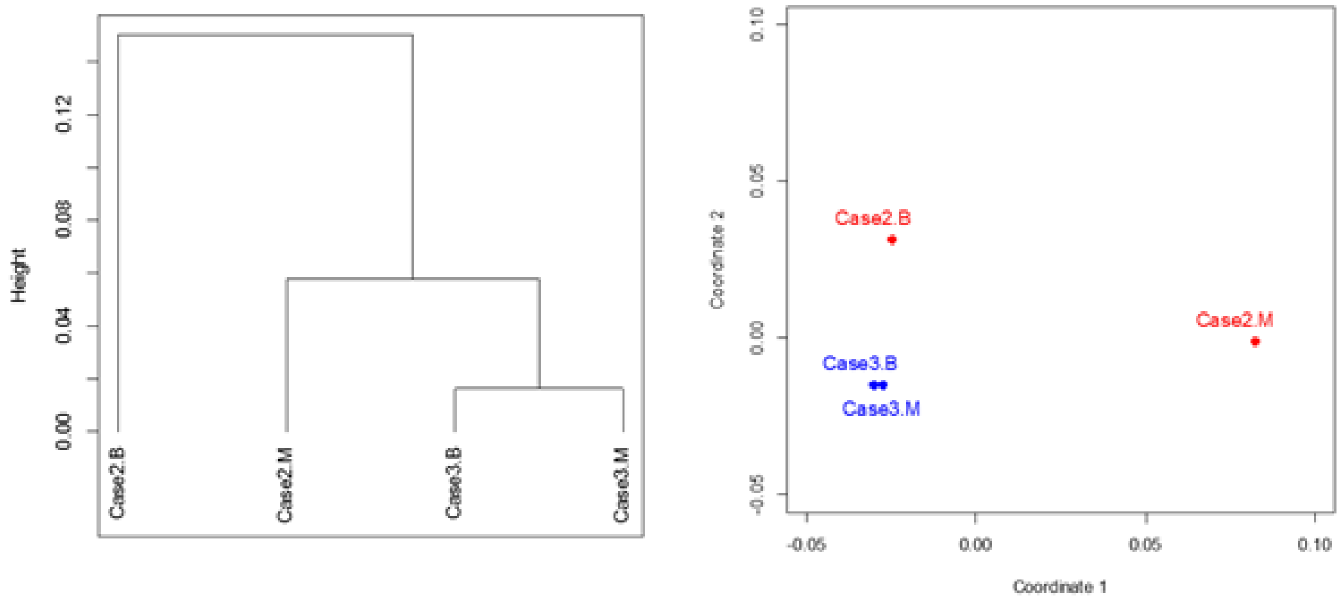

A. Dendrogram *B. Multi-Dimensional Scaling Plot*

Supplementary Figure 3: A dendrogram and a multi-dimensional scaling plot for gene expression profiles inferred from RNA-seq of Case 2 and Case 3.

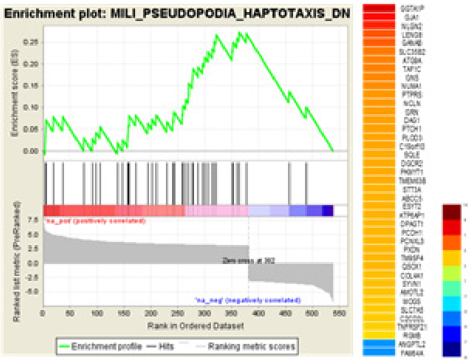

A. Gene sets enriched with genes over-expressed in malignant phenotype
(a) MILI_PSEUDOPODIA_HAPTOTAXIS_DN

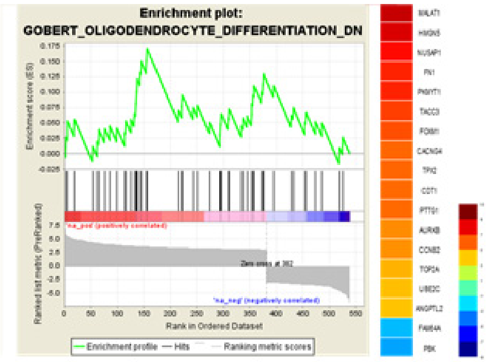

A. Gene sets enriched with genes over-expressed in malignant phenotype
(b) GOBERT_OLIGODENDROCYTE_DIFFERENTIATION_DN

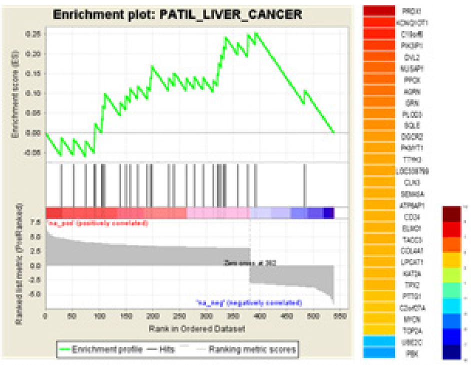

A. Gene sets enriched with genes over-expressed in malignant phenotype
(c) PATIL_LIVER_CANCER

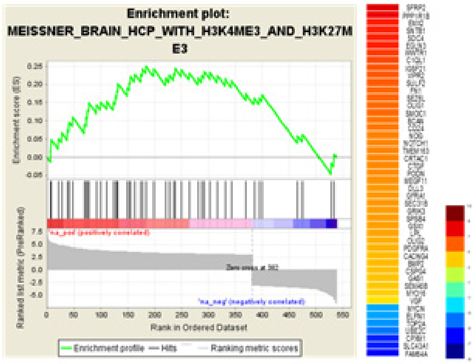

A. Gene sets enriched with genes over-expressed in malignant phenotype
(d) MEISSNER_BRAIN_HCP_WITH_H3J4ME3_AND_H3K27ME3

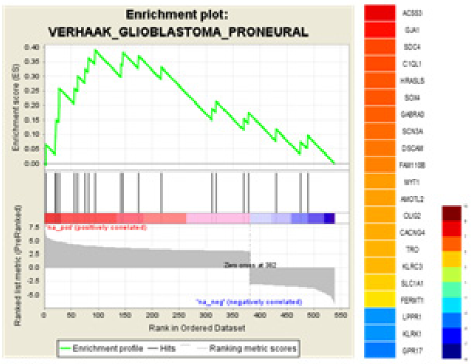

A. Gene sets enriched with genes over-expressed in malignant phenotype
(e) VERHAAK_GLIOBLASTOMA_PRONEURAL

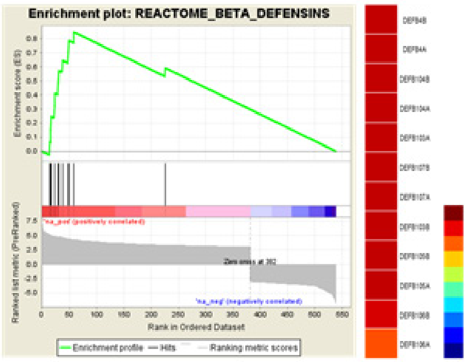

A. Gene sets enriched with genes over-expressed in malignant phenotype
(f) REACTOME_BETA_DEFENSINS

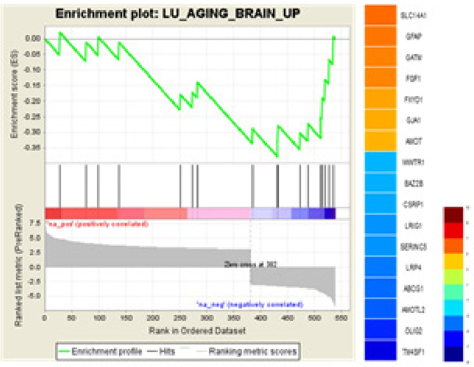

B. Gene sets enriched with genes underexpressed in malignant phenotype
(a) LU_AGING_BRAIN_UP

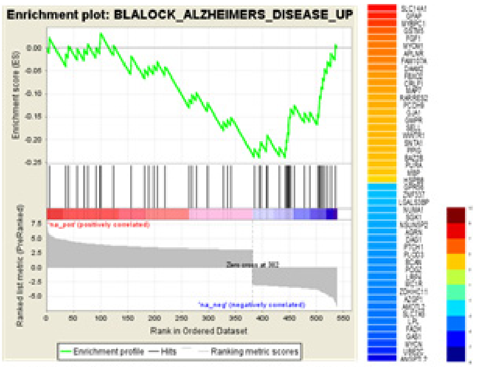

B. Gene sets enriched with genes underexpressed in malignant phenotype
(b) BLALOCK_ALZHEIMERS_DISEASE_UP

Supplementary Figure 4: The result of gene set enrichment analysis for differentially expressed genes in benign and malignant phenotype. Detailed information of gene sets are listed in Table 2.

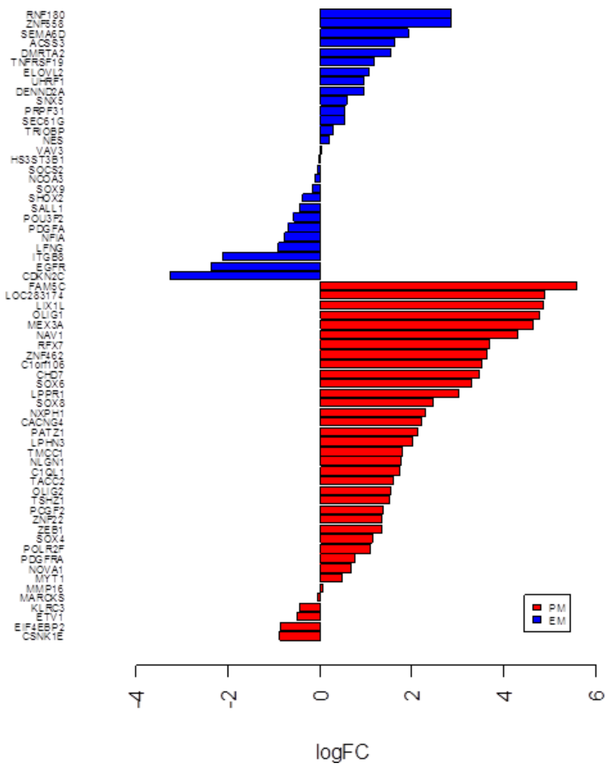

Supplementary Figure 5: Log 2 fold changes of genes between the RPKMs of benign and malignant sample of Case 2. The genes are selected from the PM (gene modules coexpressed around PDGFRA) and EM genes (gene module coexpressed around EGFR) used in the classification of glioma in Sun Y. et. al. (PNAS, 2014, 4;111(9):3538–43).

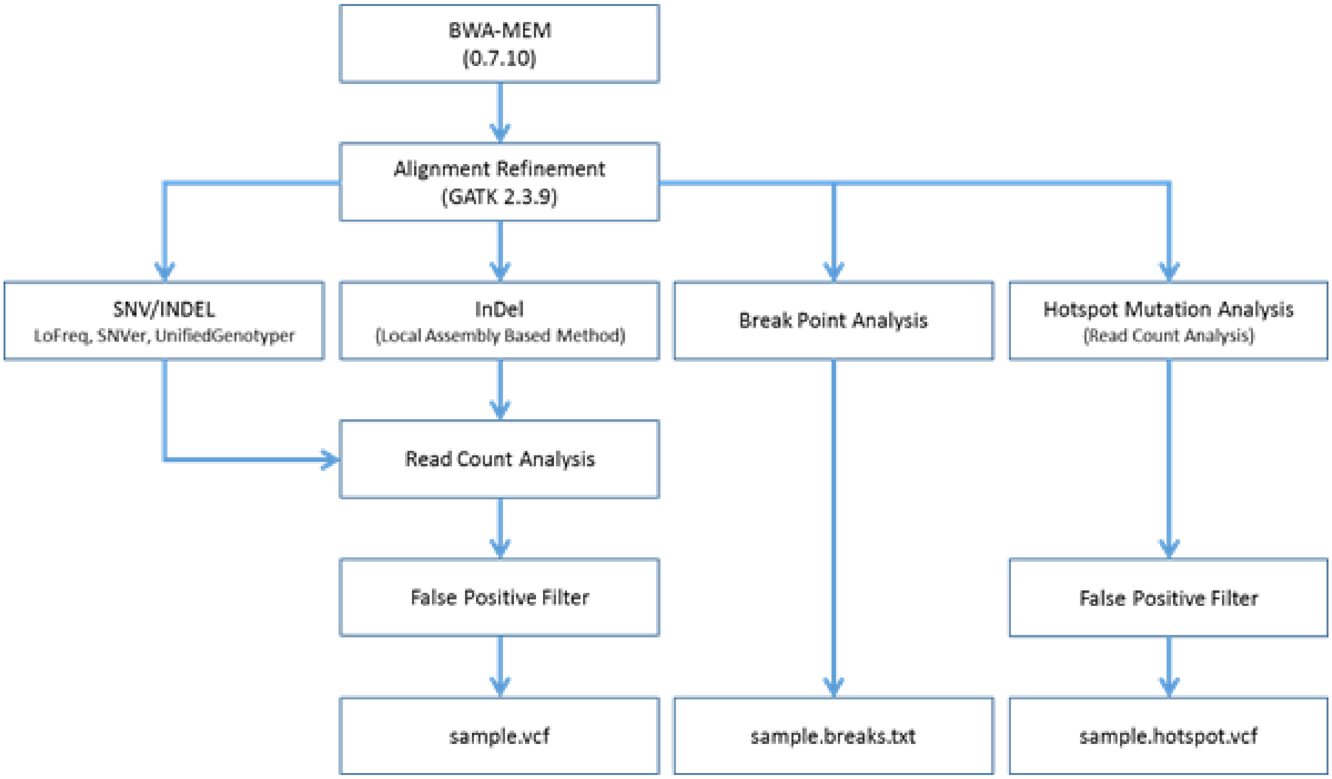

Supplementary Figure 6: Variant calling pipeline for whole exome sequencing.

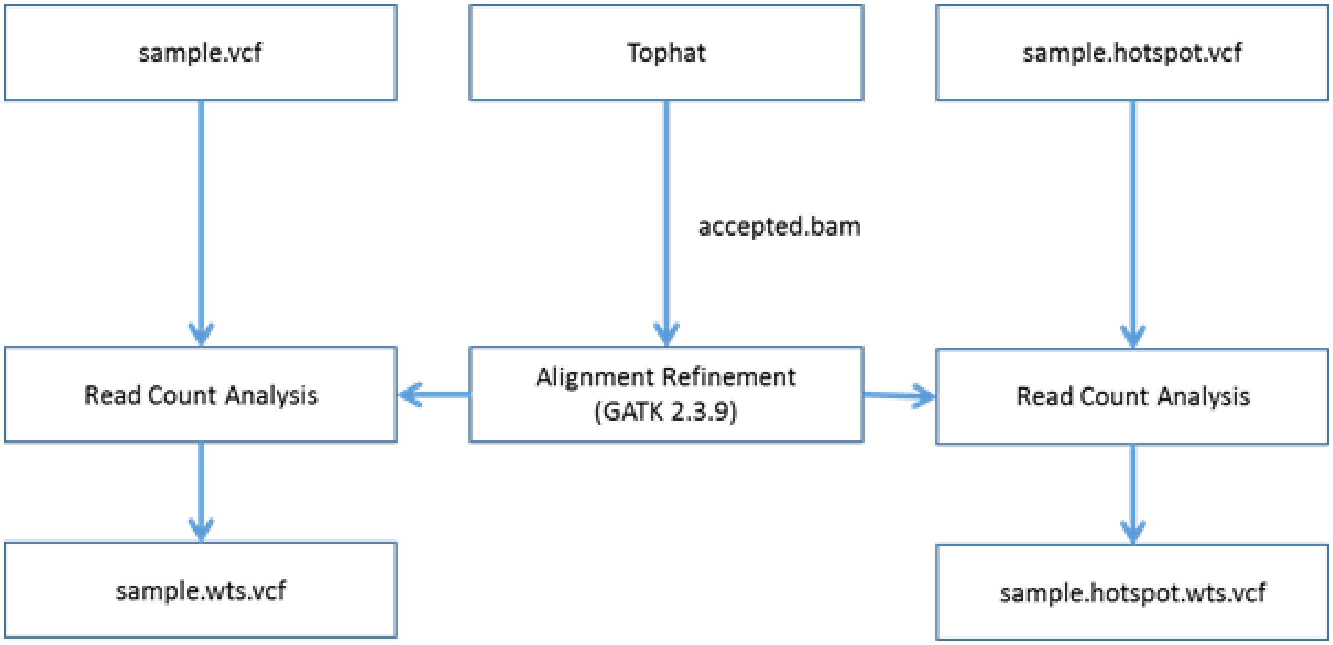

Supplementary Figure 7: Variant calling pipeline for whole transcriptome sequencing.

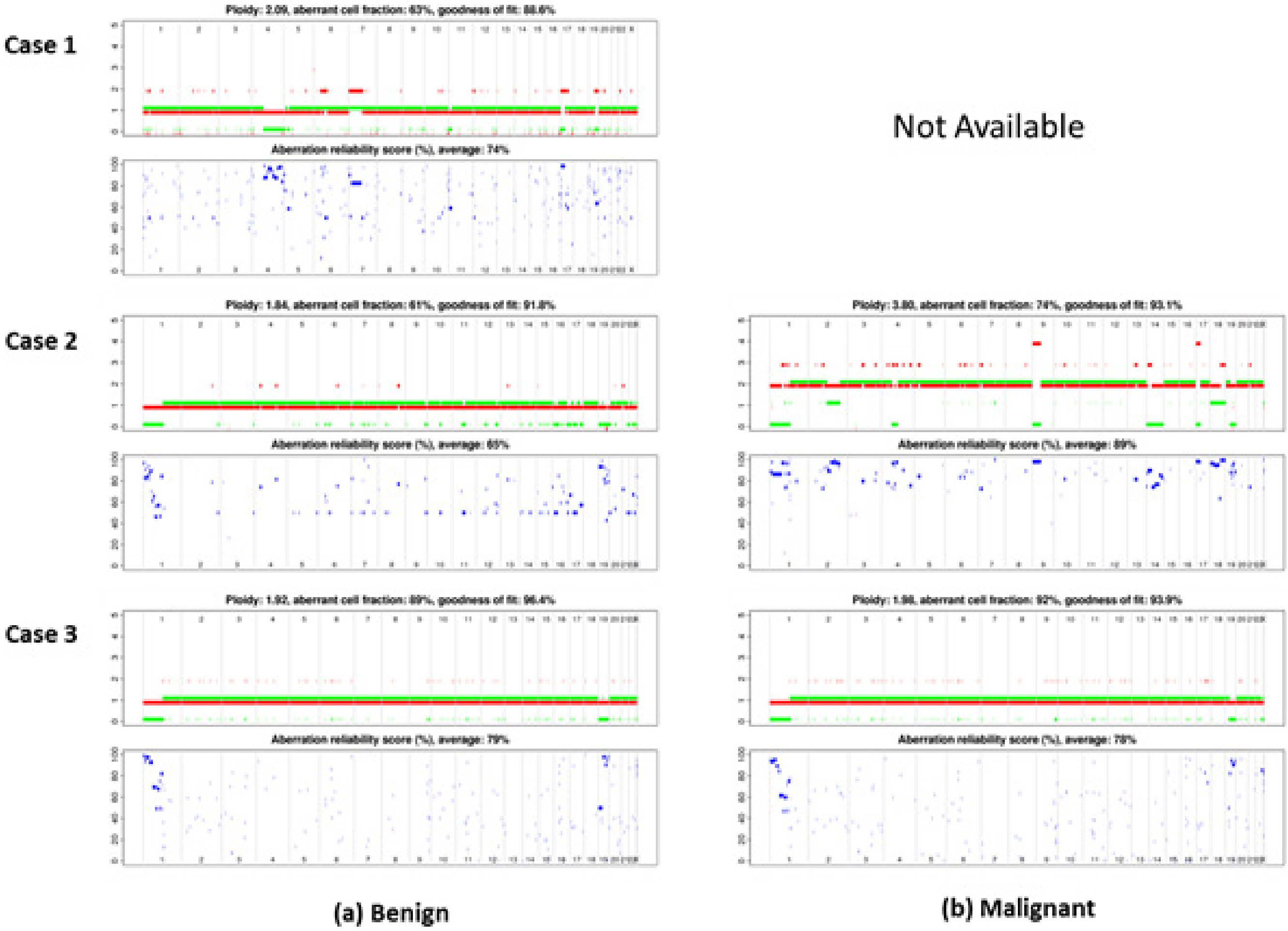

Supplementary Figure 8: Tumor ploidy and purity estimation by using ASCAT. The tumor purity of the tumor samples were eligible range of 62%–91%.

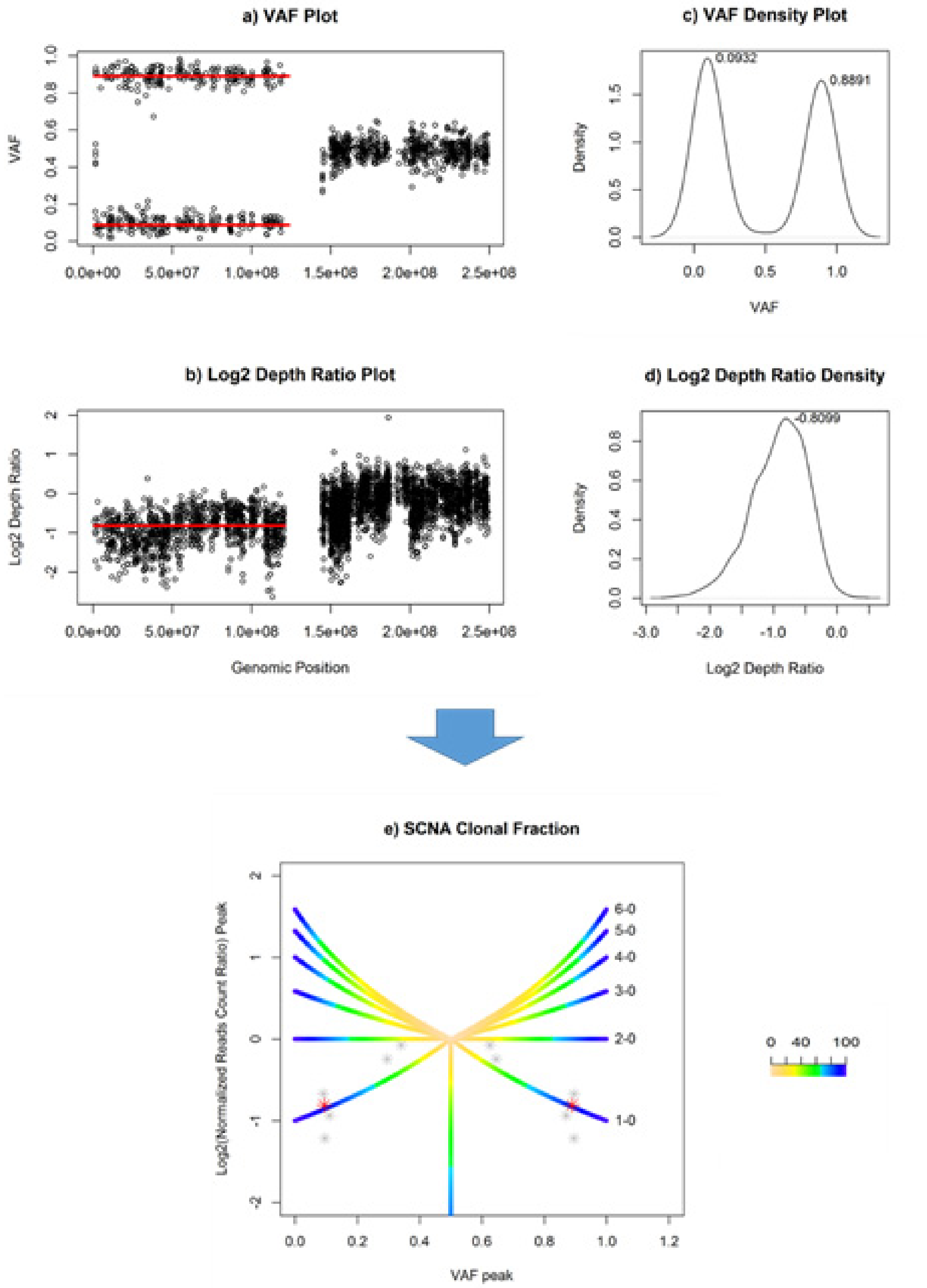

Supplementary Figure 9: Illustration of subclonal fraction estimation by SCNA. For a segment of SCNA indicated as red lines in (**a**) and (**b**), we estimate the density of variant allele frequencies (VAFs) in tumor sample for the heterozygous SNPs determined from matched normal sample (**c**) and density of log2 depth ratio between the pair of the samples (**d**) After then, we calculate the subclonal fraction with the peak values of VAF and log2 depth ratio by assuming copy number status. We can assume the copy number status of the SCNA by locating the peak values in the SCNA clonal fraction plot (**e**) The lines in e) shows theoretical location of peak values when the copy number status and the subclonal fraction are assumed. The assumed allele specific copy number status are represented at the end of lines. For example "1-0" means 1 copy loss, and "2-0" means copy neutral loss of heterozygous. The colors the lines represents the assumed subclonal fraction from 0% to 100%. The positions of the exemplar SCNA in the plot e) is denoted as red stars.

Supplementary Table 1: Stratified gene grouping system of somatic copy number alteration and somatic mutation for clonal estimation model

Group	Benign	Malignant	Description
Copy number alteration			
S0	no-alteration	no-alteration	group of genes with no change in copy number
S1.n*	deletion	deletion	group of genes with housekeeping deletion throughout the tumor lineage
S2.n*	deletion	no-deletion	group of genes undergone natural selection during the tumor progression
S3.n*	no-deletion	deletion	group of genes newly altered during the tumor progression
Somatic mutation			
M1.n*	mutation	mutation	group of genes with housekeeping somatic mutation throughout the tumor lineage
M2.n*	mutation	wild-type	group of genes undergone natural selection during the tumor progression
M3.n*	wild-type	mutation	group of genes newly mutated during the tumor progression

**n* = 1,2,3,… (in order from biggest to smallest fraction of genes with deletion or mutation)

Supplementary Table 2: Lower grade glioma cases which have IDH1 mutation from TCGA dataset for validation

1p19q status	Histological grade	Number of cases	Case IDs
Codeletion	Grade 2	45	TCGA-HT-7695, TCGA-HT-7608, TCGA-DB-A64L, TCGA-DB-A64Q, TCGA-DB-A64V, TCGA-E1-5319, TCGA-EZ-7264, TCGA-HW-7487, TCGA-E1-5318, TCGA-HT-7480, TCGA-HT-7681, TCGA-CS-6668, TCGA-DB-5278, TCGA-DB-A4XA, TCGA-DB-A64R, TCGA-DB-A64U, TCGA-DU-5870, TCGA-DU-6400, TCGA-DU-7294, TCGA-FG-5964, TCGA-FG-7634, TCGA-FG-7641, TCGA-FG-A60K, TCGA-HT-7875, TCGA-HT-7877, TCGA-HT-8012, TCGA-HW-7491, TCGA-DU-8164, TCGA-CS-5390, TCGA-DU-5849, TCGA-HT-7467, TCGA-HT-7481, TCGA-HT-A615, TCGA-FG-8187, TCGA-HT-7605, TCGA-DU-7009, TCGA-DB-5279, TCGA-DB-A4XH, TCGA-DU-5874, TCGA-HT-7607, TCGA-HT-7692, TCGA-HT-7881, TCGA-HT-8010, TCGA-HW-7486, TCGA-HW-7495
	Grade 3	34	TCGA-HT-8105, TCGA-DH-5144, TCGA-CS-5394, TCGA-DB-5274, TCGA-DU-8168, TCGA-HT-7471, TCGA-HT-7616, TCGA-HT-7677, TCGA-HT-7694, TCGA-FG-7638, TCGA-DH-5141, TCGA-DB-A64P, TCGA-DB-A64W, TCGA-DU-6393, TCGA-DU-7018, TCGA-DU-7302, TCGA-E1-5311, TCGA-FG-5962, TCGA-HT-7468, TCGA-HT-A4DV, TCGA-HT-A5R9, TCGA-IK-8125, TCGA-DU-7300, TCGA-DU-6394, TCGA-DU-6397, TCGA-HT-7620, TCGA-CS-5396, TCGA-DB-A4XG, TCGA-DU-6410, TCGA-FG-8186, TCGA-HT-7687, TCGA-HT-7856, TCGA-HT-7874, TCGA-HT-8109

Intact	Grade 2	69	TCGA-DU-6401, TCGA-CS-6667, TCGA-HT-7472, TCGA-HT-7610, TCGA-DB-5280, TCGA-DB-A4XC, TCGA-DU-5853, TCGA-DU-5871, TCGA-DU-5872, TCGA-DU-6395, TCGA-DU-6407, TCGA-DU-7007, TCGA-DU-7008, TCGA-DU-7015, TCGA-DU-7301, TCGA-DU-7306, TCGA-DU-8166, TCGA-DU-8167, TCGA-DU-A5TR, TCGA-DU-A5TU, TCGA-E1-5322, TCGA-FG-5965, TCGA-FG-6689, TCGA-FG-6690, TCGA-FG-8182, TCGA-FG-8188, TCGA-FG-A4MT, TCGA-FG-A4MX, TCGA-FG-A4MY, TCGA-HT-7473, TCGA-HT-7474, TCGA-HT-7476, TCGA-HT-7478, TCGA-HT-7482, TCGA-HT-7483, TCGA-HT-7485, TCGA-HT-7603, TCGA-HT-7604, TCGA-HT-7611, TCGA-HT-7676, TCGA-HT-7689, TCGA-HT-7693, TCGA-HT-7858, TCGA-HT-7873, TCGA-HT-7880, TCGA-HT-7902, TCGA-HT-8013, TCGA-HT-8018, TCGA-HT-8108, TCGA-HT-A5R5, TCGA-HT-A5RB, TCGA-HT-A614, TCGA-HW-7489, TCGA-HW-7490, TCGA-IK-7675, TCGA-P5-A5EW, TCGA-P5-A5F1, TCGA-CS-4938, TCGA-DB-A4X9, TCGA-FG-6691, TCGA-FG-A60J, TCGA-HT-7602, TCGA-HT-7606, TCGA-HT-7884, TCGA-DU-A5TS, TCGA-HT-A616, TCGA-CS-4944, TCGA-FG-8189, TCGA-HT-8113
	Grade 3	56	TCGA-FG-7636, TCGA-CS-6665, TCGA-DB-A4XF, TCGA-DU-6408, TCGA-CS-4942, TCGA-CS-4943, TCGA-CS-6666, TCGA-DB-5273, TCGA-DB-5275, TCGA-DB-5281, TCGA-DB-A4XB, TCGA-DB-A4XD, TCGA-DB-A4XE, TCGA-DH-5142, TCGA-DH-A66B, TCGA-DU-5851, TCGA-DU-5855, TCGA-DU-6396, TCGA-DU-7298, TCGA-DU-7304, TCGA-DU-7309, TCGA-DU-8163, TCGA-DU-A5TP, TCGA-DU-A5TW, TCGA-E1-5302, TCGA-E1-5303, TCGA-E1-5304, TCGA-E1-5305, TCGA-E1-5307, TCGA-FG-8185, TCGA-FG-8191, TCGA-FN-7833, TCGA-HT-7470, TCGA-HT-7475, TCGA-HT-7686, TCGA-HT-7688, TCGA-HT-7690, TCGA-HT-7855, TCGA-HT-7879, TCGA-HT-8114, TCGA-HT-8563, TCGA-CS-5393, TCGA-CS-6290, TCGA-DB-5277, TCGA-DH-5143, TCGA-DU-6542, TCGA-DU-7010, TCGA-DU-7019, TCGA-DU-7299, TCGA-HT-7479, TCGA-HT-7601, TCGA-HT-7609, TCGA-HT-8106, TCGA-HT-8111, TCGA-DB-5276, TCGA-HT-7684
	Grade 4	12	TCGA-06-0128, TCGA-14-4157, TCGA-14-1456, TCGA-06-0129, TCGA-06-2570, TCGA-06-6389, TCGA-02-2483, TCGA-15-1444, TCGA-19-2629, TCGA-26-1442, TCGA-27-2521, TCGA-32-4208

Supplementary Table 3: The result of RNA-seq

	Case 2		Case 3	
	Benign	Malignant	Benign	Malignant
Coverage				
Total reads	58,867,728	66,013,344	58,198,324	57,650,096
Reads map to +	26,160,089	29,492,007	26,008,229	26,223,402
Reads map to –	25,356,906	29,228,973	26,086,444	26,067,776
Multiple mapped reads	1,736,519	2,218,162	1,246,583	1,244,881
Percent of reads mapped	90.5%	92.3%	91.7%	92.9%
Fold change (FC) analysis				
Up-regulated in malignant phenotype				
log2(FC) > 3	382		8	
log2(FC) > 4	85		5	
log2(FC) > 5	19		3	
Down-regulated in malignant phenotype				
log2(FC) < – 3	157		3	
log2(FC) < – 4	33		0	
log2(FC) < –5	13		0	

Supplementary Table 4: Ploidy and purity estimation of tumor samples with SNP array by applying ASCAT

Case	Benign			Malignant		
	Ploidy	Purity	Goodness -of-Fit	Ploidy	Purity	Goodness-of-Fit
Case1	2.09	63%	88.6%	N/A	N/A	N/A
Case2	1.84	61%	91.8%	3.80	74%	93.1%
Case3	1.92	89%	96.4%	1.98	92%	93.9%
